# Examination of Anti-Inflammatory Effects After Propionate Supplementation in the R6/2 Mouse Model of Huntington’s Disease

**DOI:** 10.3390/ijms26073318

**Published:** 2025-04-02

**Authors:** Jennifer König, Alina Blusch, Oluwaseun Fatoba, Ralf Gold, Carsten Saft, Gisa Ellrichmann-Wilms

**Affiliations:** 1 Department of Neurology, St. Josef-Hospital, Ruhr-University Bochum, 44791 Bochum, Germany; 2 Department of Physiology and Pathophysiology, Center of Biomedical Education and Research (ZBAF), Faculty of Health, School of Medicine, Witten/Herdecke University, 58453 Witten, Germany; 3 Brain Disease Biomarker Unit, Wallenberg Neuroscience Center, Department of Experimental Medical Science, Lund University, BMC A10, 221 84 Lund, Sweden; 4 Department of Molecular Neuroscience, Graduate School of Medicine, Osaka University, Suita 565-0871, Japan; 5 Faculty of Health, School of Medicine, Chair of Neurology II, Witten/Herdecke University, 58448 Witten, Germany

**Keywords:** Huntington’s disease, neuroinflammation, propionate

## Abstract

Huntington’s disease is a progressive, untreatable neurodegenerative disorder caused by a mutation in the Huntingtin gene. Next to neurodegeneration, altered immune activation is involved in disease progression. Since central nervous system inflammation and dysfunction of immune cells are recognized as driving characteristics, immunomodulation might represent an additional therapeutic strategy. Short-chain fatty acids were known to have immunomodulatory effects in neuroinflammatory diseases, such as multiple sclerosis. In this study, R6/2 mice were treated daily with 150 mM propionate. Survival range, body weight, and motor abilities were monitored. In striatal and cortical samples, neuronal survival was analyzed by immunofluorescence staining of NeuN-positive cells and expression levels of BDNF mRNA by real-time polymerase chain reaction. As inflammatory marker TNFα mRNA and IL-6 mRNA were quantified by rtPCR, iNOS-expressing cells were counted in immunologically stained brain slides. Microglial activation was evaluated by immunofluorescent staining of IBA1-positive cells and total IBA1 protein by Western Blot, in addition, SPI1 mRNA expression was quantified by rtPCR. Except for clasping behavior, propionate treatment did neither improve the clinical course nor mediated neuronal protection in R6/2 mice. Yet there was a mild anti-inflammatory effect in the CNS, with (i) reduction in SPI1-mRNA levels, (ii) reduced iNOS positive cells in the motor cortex, and (iii) normalized TNFα-mRNA in the motor cortex of propionate-treated R6/2 mice. Thus, Short-chain fatty acids, as an environmental factor in the diet, may slightly alleviate symptoms by down-regulating inflammatory factors in the central nervous system. However, they cannot prevent clinical disease progression or neuronal loss.

## 1. Introduction

### 1.1. Huntington’s Disease

Huntington’s disease (HD) is a neurodegenerative disease with severe symptoms during lifespan. The disease is caused by prolonged CAG-repeat (≥36) in exon1 of the IT15 Huntingtin (Htt) gene. The mutation generates toxic full-length HTT transcripts, but also HTT exon1 fragments, discussed to be highly pathogenic, both leading to aggregation and interference with numerous signaling pathways. Incomplete splicing in Huntington’s disease patients [1,2] produces the pathogenic exon 1 Htt protein. Neuronal structures in the basal ganglia and cortex are the most affected areas showing early dysfunction and cell death [2]. This results in several symptoms related to motor, cognitive, and psychological impairments. The leading symptoms are movement disturbances, preceding immobility, and persistent weight loss [3]. Additionally, depression, anxiety, personality change, difficulties in cognition, and dementia might occur during life span [4]. The onset of symptoms predominantly appears in adult individuals with an average age of 40 years, but also juvenile and late-onset cases are known, depending on the number of CAG repeats [5,6,7]. Most patients die 17–20 years after symptom onset [8].

The mutation of the Htt protein was identified as the cause of the disease in 1993 [9]. Since then, research has been conducted to find ameliorative treatments, and several strategies to lower Htt are currently under investigation [10,11]. However, the treatment of HD patients is still limited to symptomatic management with drugs such as neuroleptics or dopamine-depleting agents [12,13,14,15]. This study aims to investigate the effect of the natural compound propionate on symptoms in R6/2 mice.

### 1.2. Neuroinflammation in HD

Neuroinflammation is characterized by the extended expression of pro-inflammatory cytokines, chemokines, and oxidative stress, whereas microglia and astrocytes serve as major sources next neurons [16]. Microglia prevent neurological harm in a healthy system, aberrant activation occurs as a common feature across several neurodegenerative diseases [17,18,19,20,21]. In HD individuals activated microglia already occur in the preclinical stage and persist during disease progression. Activated microglia correlate with neuronal dysfunction in the striatum, resembling a chronic inflammatory state [22,23]. Increased levels of Interleukin-17 (IL-17), a pro-inflammatory cytokine secreted by activated T-cells as well as an increased prevalence of IL-17–producing Th17.1 cells in the CSF of premanifest HD mutation carriers further underlines inflammation as an early and important part the pathophysiology in HD [24]. Additionally, elevated pro-inflammatory cytokine levels are measured in the plasma of HD individuals, which might contribute to neuroinflammation and disease progression [25]. An imbalance of inflammatory mediators like tumor necrosis factor (TNF)α and Interleukin-6 (IL-6) might have neurotoxic effects and might contribute to the severity of cognitive and motor coordination dysfunction [26,27]. mRNA expression profile changes in the human prefrontal cortex further support the role of inflammation and the functional significance of non-neuronal involvement in HD pathogenesis, suggesting anti-inflammatory strategies as an important chance for the treatment of HD. Consequently, various preclinical research on immunomodulatory agents to slow or ameliorate neurodegeneration have been conducted in the last years [28,29,30,31,32].

In patients, treatment with Laquinimod leads to reduced caudate volume loss if compared with placebo treatment [33]. Potentially positive effects of immunomodulatory therapies on disease progression in Huntington’s disease were found also in a study comparing the course of the disease in patients suffering from both, HD and autoimmune demyelinating diseases of the central nervous system and patients suffering from HD only [34].

### 1.3. Propionate

Short-chain fatty acids (SCFA) are key bacterial fermentation products in the human intestine. Various bacteria of different families enzymatically break down plant-derived polysaccharides and produce SCFA, mainly acetate, propionate, and butyrate. From the intestine, acetate, and propionate are transported to the body by blood flow and show systemic effects, whereas the concentration declines towards the periphery [35,36]. The ratio and total concentration of SCFA depend on diet and the composition of the intestinal microbiome. They are of special interest since they are recognized as a systemic regulator of individual health. SCFAs in pharmacological applications were observed more than 20 years ago during the treatment of intestinal inflammation when a deficiency of SCFA was identified as a common feature in colitis or inflammatory bowel disease patients [37,38,39]. Interestingly, the relevance of the intestinal microbiome and SCFA was extended to neurodegenerative diseases, underlining the systemic effect of environmental factors on health and disease. Duscha et al. revealed low SCFA concentration, especially of propionate, in serum and fecal samples of people with Multiple sclerosis (MS), due to poor intestinal microbiota diversity and colonization due to chronic inflammation. Strikingly, the supplementation of propionate in patients significantly reduced relapse rate and neurodegeneration [40]. In HD, less is known about the intestinal microbiome and the metabolism of SCFA. A study by Wasser et al. revealed a reduced variety in microbiota compared to non-mutation carriers [41]. Microbiome composition in male individuals significantly differed. The effect of SCFA on symptoms and disease progression in HD is still unknown and may serve as a focus of future research. Considering studies of the last decade, propionate is assumed to mediate an anti-inflammatory effect under pro-inflammatory conditions, thus restoring homeostasis in pathological conditions [42,43]. In this experimental study, we investigated whether propionate treatment can modulate disease progression in the R6/2 mouse model of HD.

## 2. Results

### 2.1. Propionate Treatment Did Not Affect Signs of Clinical Progression but Ameliorated Clasping Behavior in R6/2 Mice

**Figure 1:**
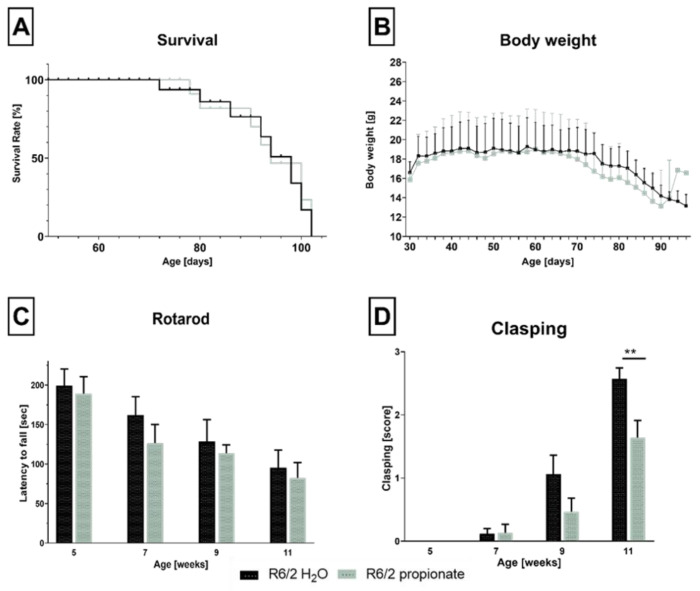
Propionate treatment did not affect signs of clinical progression but ameliorated clasping behavior in R6/2 mice. (**A**). Kaplan-Meier survival curve. Median survival for control mice (*n* = 10) was 89 days and for treated mice (n = 14) 88 days. No prolonged survival rate was observed for propionate-treated R6/2 mice. Kaplan-Meier analysis with a log-rank test was performed as a test for significance. (**B**). R6/2 mice gain weight until the age of 50 days and start losing weight at 65 days of age (R6/2-H_2_O 18.76 ± 0.176, R6/2-propionate 18.69 g ± 0.185). No difference between both groups was measured. Due to the sudden natural death of animals during survival study, *n*-number in both groups varies R6/2-propionate n ≥ 14, R6/2-H_2_O n ≥ 10. Unpaired *t*-test with individual variance for each row. (**C**). Rotarod performance in the survival group. Performance declined with disease progression in treated and control mice. R6/2-propionate n ≥ 14, R6/2 H_2_O n ≥ 10. Two-way ANOVA with Bonferroni’s multiple comparisons test. (**D**). Clasping was less severe in propionate-treated mice over time, with significant differences at eleven weeks of age (** *p* = 0.003) compared to water-treated mice. R6/2-propionate n ≥ 18, R6/2 H_2_O n ≥ 15. Two-way ANOVA with Bonferroni’s multiple comparisons test. Data are shown as mean ± standard error of the mean (SEM).

During life span, R6/2 mice undergo progressive motoric deficits and body weight loss starting from the pre-symptomatic stage over the mild symptomatic stage until the severe symptomatic stage [44]. The clinical course of disease progression was observed in treated and untreated R6/2 mice starting in a pre-symptomatic stage at the age of 30 days. Kaplan-Meyer-Survival analysis (Figure 1A) revealed a median survival age of 93 days for untreated mice and 88 days for propionate-treated mice, with no significant differences between groups. Focusing on body weight (Figure 1B), R6/2 mice showed the typical age-dependent body weight plateau at 40 days which further declined significantly at 70 days of age. Propionate treatment did not rescue disease-related body weight loss (Figure 1B).

Furthermore, we investigated the effects of propionate on motor coordination and balance. Propionate treatment had no influence and performance declined in the treated group (Figure 1C). R6/2 mice develop a clasping phenotype in later disease stages, which is usually scored to show symptom progression related to dyskinesia [45]. Clasping behavior was first observed in seven-week-old mice and worsened with disease progression (Figure 1D). At eleven weeks, all mice displayed worsening clasping behavior. However, propionate-treated mice developed a significantly less severe phenotype compared to the control group at this age (Figure 1D). In addition, mice were tested for their active behavior in the open field. Propionate treatment did not affect parameters such as active time, speed, or measured distance. 

**Figure 2:**
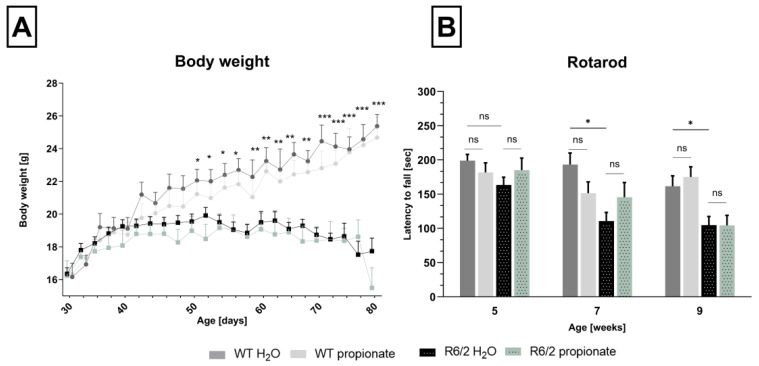
Wild-type control group in comparison to R6/2 mice. (**A**). Difference in body weight (wildtype control mice vs. R6/2 mice) became significant starting at the age of 50 days (Mean at 50 days of age R6/2-H_2_O 18.76 ± 0.176, R6/2-propionate 18.69 g ± 0.185, WT-H_2_O 21.84 g ± 0.5, WT-propionate 21.10 g ± 0.45, WT-H_2_O vs. R6/2-H_2_O * *p* = 0.023, WT-H_2_O vs. R6/-propionate * *p* = 0.048) With increasing age, differences in weight development become more pronounced (** *p* < 0.01, *** *p* < 0.001.) (**B**). Rotarod performance significantly declined in water-treated R6/2 mice at the age of 7 weeks (WT H_2_O vs.R6/2 H_2_O * *p* = 0.0004) and 9 weeks (WT H_2_O vs. R6/2 H_2_O * *p* = 0.008). Propionate treatment did not influence motor performance (ns = not significant). Two-way ANOVA with Bonferroni’s multiple comparisons or multiple unpaired *t*-tests with Bonferroni’s correction was performed, respectively. Data are expressed as mean ± SEM.

Experiments were preceded with a second experimental group also containing wild-type littermates as the control group for bodyweight development and rotarod analysis. In line with previous results, propionate treatment did not show any effect, neither in R6/2 mice nor in wild-type littermates. R6/2 mice, treated and untreated, significantly declined in body weight and rotarod performance compared to healthy control mice from 7 weeks of age (Figure 2). For further experiments, these animals were sacrificed for analysis of central markers for neurodegeneration and inflammation.

### 2.2. Neuronal Decline Occurs in Propionate-Treated R6/2 Mice

**Figure 3:**
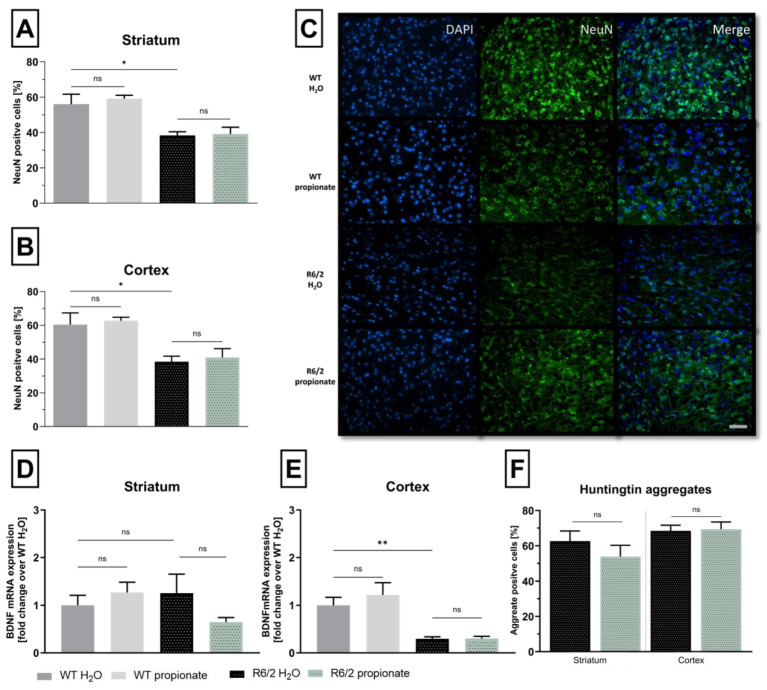
Neuronal decline occurs in propionate-treated R6/2 mice. (**A**,**B**). Brain slides of 12-week-old mice were stained for NeuN-positive cells. Counted cells were normalized to the total amount of cells visualized with DAPI. Wild-type littermates had significantly more neurons in the striatum and cortex than R6/2 mice. Striatum: WT-H_2_O vs. R6/2-H_2_O * *p* = 0.012. Cortex: WT-H_2_O vs. R6/2-H_2_O * *p* = 0.016. WT-H_2_O n = 4, WT-propionate n = 4, R6/2-H_2_O n = 5, R6/2-propionate n = 5. Data were analyzed with one-way ANOVA and Bonferroni’s post hoc test for multiple comparisons. Compared groups are indicated in the figures as significant (* *p* > 0.05) or not significant (ns). (**C**). Representative fluorescence images of cortical brain slides stained for NeuN in green and DAPI in blue. Scale bar represents 50 µm. (**D**,**E**). BDNF-mRNA expression in 12-week-old mice. Striatal levels in wild-type littermates are comparable to levels in transgenic mice. Cortical levels in R6/2 mice showed a significant decrease and propionate treatment had no protective effect. Cortex: WT-H_2_O vs. R6/2-H_2_O ** *p* = 0.004. WT-H_2_O n = 4, WT-propionate n = 8, R6/2-H_2_O n = 8, R6/2-propionate n = 6. One-way ANOVA with Bonferroni’s test for multiple comparisons. Groups compared are indicated in the figures as significant or not significant (ns). (**F**). Number of aggregate-positive cells, either in the striatum or motor cortex, were not affected by propionate treatment. Significance was calculated using the two-tailed, unpaired Student’s *t*-test. R6/2-propionate n = 9, R6/2-H_2_O n = 9. All data in this figure are presented as mean ± SEM.

Propionate has been shown to exert neuroprotective potential in different models for diseases like Alzheimer’s disease (AZ) [46] or MS [42]. Therefore, neuronal marker (NeuN) immunostaining and mRNA levels of brain derived neurotrophic factor (BDNF) were examined. In R6/2 mice, NeuN immunostaining revealed a significant decline in the number of neurons compared to wild-type littermates. Similarly, treatment with propionate showed no protective effect on neuronal loss (Figure 3A,B). Figure 3C exemplarily displays immunofluorescent staining of neuronal marker NeuN in the cortical area of wild-type and R6/2 mice, where a reduction is evident in transgenic mice. Striatal BDNF-mRNA level remains unchanged in R6/2 mice compared with WT littermates (Figure 3D). In the cortex, however, BDNF-mRNA level decreased significantly in R6/2 mice compared with WT littermates. In line, propionate treatment failed to rescue cortical BDNF reduction in R6/2 mice (Figure 3E). Aggregation of mutated Htt in the CNS correlates with HD’s neurological symptoms [47]. We closely quantified the number of cells containing huntingtin aggregates in the striatum and the motor cortex. Both areas comprise a similar percentage of aggregate positive cells, yet propionate treatment showed no influence on aggregation revealed by EM48 immunostaining (Figure 3F).

### 2.3. Examination of Microglia Activation

**Figure 4:**
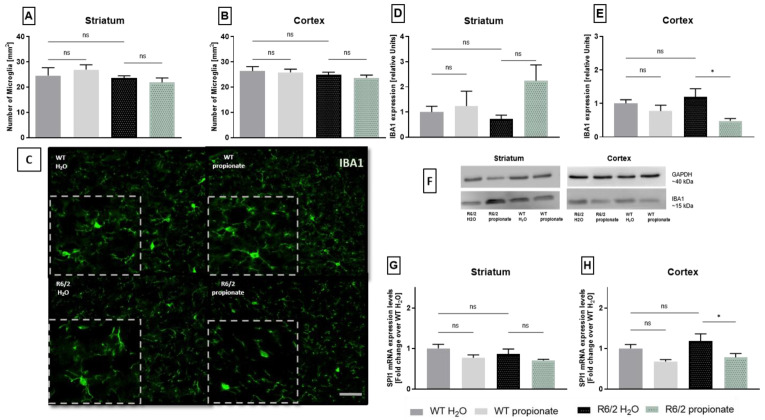
Microglia analysis in WT and transgenic mice. (**A**,**B**). Microglia cell bodies in IBA1 stained brain slides. Numbers of microglia did not change between groups (ns = not significant). WT-H_2_O n = 4, WT-propionate n = 8, R6/2-H_2_O n = 8, R6/2-propionate n = 6. One-way ANOVA with Bonferroni’s post hoc test. Groups compared are indicated in the graphs. Data are presented as mean ± SEM. (**C**). Representative images of fluorescent IBA1 staining in the cortex of mice. Equal amounts of cell bodies are stained, but fewer protrusions are seen in cortices of R6/2 mice treated with propionate. Enlarged sections of the original photographs for demonstration purposes are shown in dotted lines. Scale bar represents 50 µm. (**D**,**E**). Western Blot analysis of striatal and cortical lysates. Propionate treatment reduced IBA1 levels in R6/2 mice cortices (R6/2-H_2_O vs. R6/2-propionate * *p* = 0.0273). WT-H_2_O n = 3, WT-propionate n = 6, R6/2-H_2_O n = 6, R6/2-propionate n = 6. One-way ANOVA with Bonferroni’s test for multiple comparisons. Groups compared are indicated in the graphs. Data are presented as mean ± standard deviation. (**F**). IBA1 and GAPDH expression levels in striatal and cortical samples detected in Western Blot experiments. (**G**,**H**). In striatal samples, SPI1-mRNA was equally expressed in all groups. Also, in cortical samples of R6/2 mice mRNA levels were not significantly upregulated. Yet, propionate treated R6/2 mice expressed significantly less SPI1-mRNA (R6/2-H_2_O vs. R6/2-propionate * *p* = 0.0352). Group comparison with one-way ANOVA and Bonferroni’s post hoc test, as indicated in the graphs. Data are presented as mean ± SEM. WT- H_2_O n = 3, WT-propionate n = 7, R6/2-H_2_O n = 5, R6/2-propionate n = 6.

To quantify a possible effect on microglia activation, the number of microglia was counted in IBA1 stained brain slides. The total number of microglia was equal in wild-type littermates and R6/2 mice. Similarly, propionate treatment did not affect the total number of microglia (Figure 4A,B). However, histological staining revealed different patterns and intensities of IBA1-positive structures, as indicated in Figure 4C.

Therefore, we proceeded to quantify whole IBA1 levels by Western blot analysis. Total IBA1 protein levels were not upregulated in R6/2 mice compared to wild-type littermates in striatal and cortical samples (Figure 4D,E). Yet, lower IBA1 expression was observed in the cortex of propionate treated R6/2 mice compared to water treated R6/2 mice. (Figure 4E). Figure 4F shows exemplary western blot bands. To further quantify microglia activation, expression of microglia-specific transcription factor PU.1/SPI1 was analyzed by rtPCR. In previous studies, SPI1 was related to inflammatory responses in HD [48]. Analysis revealed no differences in mRNA levels between striatal samples of wild-type and R6/2 mice. Also, propionate treatment showed no effect in R6/2 mice (Figure 4G). A similar pattern was observed in cortical samples, where R6/2 mice exhibited no upregulation of SPI1 mRNA expression. However, a decrease in SPI1 mRNA levels was observed in propionate-treated R6/2 mice compared to H_2_O-treated R6/2 mice (*p* = 0.0352) (Figure 4H).

### 2.4. iNOS Expression and TNFα-mRNA Levels Are Altered in Propionate-Treated R6/2 Mice

**Figure 5:**
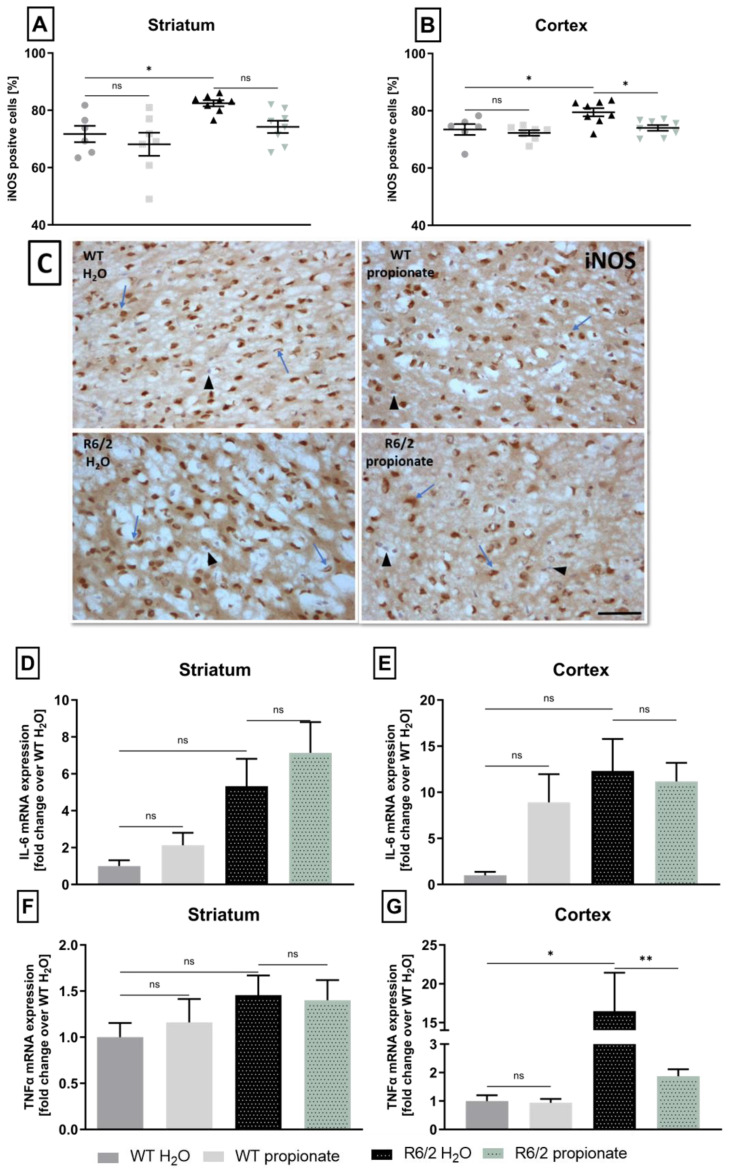
iNOS expression, as well as TNFα-mRNA levels, are reduced in the cortex of propionate-treated R6/2 mice. (**A**,**B**). Expression of iNOS in striatal and cortical CNS cells in 12-week-old mice. The striatum of R6/2 control mice comprised more iNOS-positive cells than wild-type mice (WT-H_2_O vs. R6/2-H_2_O * *p* = 0.0275). Reduction by propionate treatment was not significant (ns). In the cortex propionate treatment decreased iNOS positive cells significantly (WT-H_2_O vs. R6/2-H_2_O * *p* = 0.013, R6/2-H_2_O vs. R6/2-propionate * *p* = 0.015). WT-H_2_O n = 6, WT-propionate n = 7, R6/2-H_2_O n = 8, R6/2-propionate n = 8. One-way ANOVA with Bonferroni’s test for multiple comparisons. Group comparison was performed as indicated in the figures. Data are shown as mean ± SEM. (**C**). Representative light microscope images of cortical brain slides stained for iNOS. iNOS-positive cells are identified by DAB-immunoreaction and indicated with blue arrows. Counterstaining with Meyer’s hemalaun solution revealed iNOS negative cells by a light blue appearance as indicated with black arrowheads. Scale bar represents 100 µm. (**D**,**E**). IL-6-mRNA expression in 12-week-old mice. Striatal levels were not significantly regulated, neither in R6/2 mice nor by propionate treatment. WT-H_2_O n = 4, WT-propionate n = 10, R6/2-H_2_O n = 11, R6/2-propionate n = 10. One-way ANOVA with Bonferroni’s post hoc test. Groups compared as indicated. Data are presented as mean ± SEM. (**F**,**G**). TNFα-mRNA expression in the cortex of 12-week-old R6/2 mice was increased, whereas propionate-treated R6/2 mice mRNA levels were downregulated. (Cortex: WT-H_2_O vs. R6/2-H_2_O * *p* = 0.024, R6/2-H_2_O vs. R6/2-propionate ** *p* = 0.0045). WT-H_2_O n = 4, WT-propionate n = 8, R6/2-H_2_O n = 11, R6/2-propionate n = 8. One-way ANOVA with Bonferroni’s post hoc test. Groups compared as indicated. Data are presented as mean ± SEM.

Next, additional parameters of inflammation were examined under the influence of propionate. The expression of inducible nitric oxide synthase (iNOS), a marker for pro-inflammatory stimuli [49]. Analysis revealed an elevated percentage of iNOS-positive cells in R6/2 mice in both brain areas, the striatum and motor cortex. Interestingly, propionate treatment significantly reduced iNOS-positive cells in the motor cortex of R6/2 mice (*p* = 0.015) (Figure 5A,B). Histological stainings, exemplarily shown in Figure 5C, were analyzed for DAB-stained iNOS-positive cells (indicated by blue arrows) and iNOS-negative cells (indicated by black arrowheads), which lack DAB-staining and therefore were only counterstained with hemalaun.

Furthermore, mRNA levels of inflammatory cytokines TNFα and IL-6 were quantified, as these cytokines are potential inducers of iNOS.

For IL-6, R6/2 mice showed no significant increase compared to wild-type littermates in either the striatum or cortex (Figure 5D,E).

TNFα mRNA was equally expressed in the striatum of R6/2 and wild-type mice (Figure 5F). However, in the cortex, TNFα mRNA was highly elevated in control R6/2 mice, suggesting active inflammatory conditions in these animals. Conversely, propionate-treated R6/2 mice exhibited reduced TNFα mRNA expression levels (Figure 5G).

## 3. Discussion

HD has a monocausal origin: the CAG-repeat expansion in the huntingtin gene. However, the disease course widely varies, even with similar CAG-repeat numbers. Therefore, environmental influences are assumed to play a role in disease progression and symptom severity. Environmental factors such as diet, exercise, smoking, and infections have increasingly been considered as possible modulators [50,51]. Of those, external influences such as nutrition and the production of SCFA may contribute to the disease course. Current research focuses on the composition of the microbiome and potential changes, as its impact on SCFA in HD patients to further shed light on disease onset and clinical progression [52]. First studies show in R6/1 mice, the composition of intestinal microbiota was altered before symptom onset, potentially playing a role in disease onset [53]. A study with MS patients revealed decreased propionate levels in serum and fecal samples and a substitution of propionate was proven to be beneficial in terms of neuroprotective and anti-inflammatory [40]. Therefore, the substitution of propionate displays a treatment option to be investigated in models for HD.

This study is one of the first to examine the relevance of propionate on CNS in HD using the R6/2 mouse model. We aimed to observe the influence of propionate on typical signs of disease progression. Herein, the effect of clinical disease course, and its influence on neurodegeneration, and neuroinflammation was quantified. Primary findings did not show significant amelioration of the clinical disease course (body weight, rotarod, open field, or survival) nor rescued neuronal loss (NeuN staining, BDNF mRNA levels) in the brain of R6/2 mice.

Primarily, HD is a neurodegenerative disease marked by aggregation of mutated Htt mainly in neurons and associated with neuronal dysfunction followed by severe neuronal loss. The formation of aggregates is followed by neuronal degeneration which has been directly correlated with disease progression, including the onset and development of motor symptoms [54]. Verse visa, neuroprotection in HD results in ameliorated disease progression. To test whether propionate might have a neuroprotective effect, this study examined aggregate formation and neuronal survival in R6/2 mice by EM48 staining and NeuN staining, respectively. In addition, neuronal support was examined by measuring BDNF, since it exerts a neuroprotective effect as an essential survival factor for neurons. In HD, BDNF production is impaired, resulting in a lack of cortico-striatal BDNF supply, thus playing a crucial role in neuronal loss in the striatum [55,56,57]. In the present study mutated Htt-aggregate formation and neuronal loss were evident in the striatum and cortex of R6/2 mice despite propionate treatment. In addition, cortical BDNF production was strikingly reduced. Together, the results demonstrate that propionate cannot rescue neuronal survival. Nonetheless, a reduced clasping score was observed in the advanced stage of the disease. The relationship between ameliorated clasping behavior and progressive neuronal loss remains to be elucidated. One potential approach is to consider the CNS as a network aiming to stabilize neuronal functionality rather than focus on neuronal survival in terms of symptom progression. In this CNS network, a prominent fraction consists of immune cells such as microglia, which support neuronal function in various ways. Under physiological conditions, microglia continuously survey the brain environment, removing cellular debris and maintaining the balance of neuronal circuits, and are referred to as being in a resting state [58]. In HD pathology, microgliosis, and chronic microglial activation are major features of the neuroinflammatory process, where microglia lose their supportive functions and gain a pro-inflammatory phenotype [59,60,61,62]. Microgliosis is a complex process characterized by various inflammatory stages and several indicators such as morphological changes, proliferation, upregulation of marker proteins, and the production of inflammatory cytokines [58]. Particularly in neuroinflammatory diseases when chronic inflammation occurs, these indicators are reported, e.g., for AD, MS, and HD. Therefore, we assessed different parameters of microglial activation.

Starting with IBA1-stainings, we observed no significant change in microglia numbers in the brains of R6/2 mice, thus excluding altered proliferation in these mice. Notable, no upregulation of IBA1 in untreated R6/2 mice was examined, suggesting a lack of microglial activation. However, fluorescent staining suggested a decrease in IBA1-positive patterns in propionate-treated R6/2 mice. Indeed, further quantification by Western blot revealed a significant decrease in IBA1 total protein level in cortical samples from propionate-treated R6/2 mice, but not in the striatum.

Interpretation of this finding requires consideration of several factors. In the context of HD, IBA1 upregulation is not a consistent event, since it is expression varies in R6/2 mice depending on age and brain region. For example, IBA1 upregulation in R6/2 mice is described in 15-weeks-old mice [58] but is not evident in the striatum of 11.5-weeks-old R6/2 mice [63]. Moreover, while IBA1 is an established marker for microglia, its expression is no distinguish marker for microglia activation, since it is expressed by resting as well as activated microglia. Therefore a differentiation between resting and activated microglia requires further analysis, as e.g., morphological characterization or additional molecular marker [64,65]. Consequently, the lack of elevated IBA1 expression does not necessarily indicate the absence of pro-inflammatory potential.

In the next step we analyzed SPI1 as a second marker for microglial activation. SPI1 is a marker for the lineage-specific transcription factor PU.1, which is reported to promote microglia pro-inflammatory cytokine production [48]. In HD, microglia dysfunction is an autonomous event due to interference of mutated Htt with signaling pathways including, among others, SPI1. The interaction of mutated Htt and SPI1 leads to enhanced transcriptional activation of SPI1 and promotes pro-inflammatory gene transcription and elevated cytokine production such as TNFα and IL-6 [48]. SPI1 activity in microglia during pathological neuroinflammation has also been demonstrated in cell-based models of AD. Overexpression of SPI1 drives microglia into a pro-inflammatory state by enhancing the production of pro-inflammatory cytokines, with increased levels of TNFα and iNOS being observed. Conversely, SPI1 knockdown reduces pro-inflammatory mediator expression. These findings highlight SPI1 regulation as a promising therapeutic target for modulating neuroinflammation [66]. The present results did not indicate an increased expression of SPI1 mRNA in R6/2 mice, which is consistent with the observed IBA1 levels and raises questions about the activation status of microglia in 12-week-old R6/2 mice. However, the experimental design did not assess the transcriptional activity of the SPI1 transcription factor, leaving open the question of its involvement in the production of pro-inflammatory mediators.

Nonetheless, propionate treatment significantly reduced SPI1 mRNA expression in the cortex of R6/2 mice. This result reflects the reduction in total IBA1 protein levels seen in cortical samples from propionate-treated R6/2 mice. Taken together, these findings suggest a microglia-specific immunomodulatory effect of propionate, while also highlighting the need for investigation on microglia characterization with a focus on HD. This aspect becomes even more complex considering that inflammation in Huntington’s disease (HD) is both stage- and brain region-dependent. As such, inflammatory responses and the surrounding environment are likely influenced by variations in microglial composition, which were categorized into functional clusters. These clusters emerge in an age-dependent manner in R6/2 mice and exhibit distinct differences in inflammatory profiles, phagocytic activity, and morphology [67].

Subsequent analysis of pro-inflammatory markers such as iNOS, and the cytokines TNFα and IL-6 was performed to further explore the inflammatory environment in the CNS and the effect of propionate in R6/2 mice. In neuroinflammation, iNOS is typically upregulated and serves as a marker for the extent of inflammation since the expression levels rise with prolonged inflammatory conditions [68]. In our experiments, iNOS was increased in the brains of R6/2 mice seen by elevated numbers of iNOS-positive cells, confirming pro-inflammatory conditions within striatal and cortical areas. However, iNOS expression was shown to be reduced by propionate in R6/2 mice, supporting its anti-inflammatory potential. In line with previous findings, this reduction was evident in the cortex, while a reduction in the striatum was not significant. Subsequently, pro-inflammatory cytokine production was analyzed. In the striatum, TNFα mRNA expression levels were consistent with the previously examined markers and showed no signs of a pro-inflammatory environment. However, in the cortex, TNFα expression was significantly elevated in R6/2 mice, indicating a pro-inflammatory environment. Treatment with propionate led to a reduction in TNFα levels, supporting previous findings that propionate exerts an anti-inflammatory effect in the cortex in R6/2 mice.

Furthermore, a potential correlation can be inferred between the reduced SPI1 mRNA and TNFα mRNA levels in the cortex of propionate-treated R6/2 mice, given SPI1’s established role in regulating the transcription of TNFα and other inflammatory genes, as mentioned before.

In addition, it is hypothesized that a reduction in TNFα action may be reflected in a decrease in iNOS-positive cells: Since TNFα is a key stimulus for iNOS expression in the inflammatory brain [69,70], and therefore, reduced TNFα-mRNA levels could result in fewer iNOS-positive cells, thus limiting inflammation and neuronal damage. Interestingly, propionate has been shown to regulate both cytokine release and iNOS levels in vitro [71], underlining a possible link between these effects. Considering microglia as the primary source of cytokines in the CNS, such as TNFα and IL-6 [72], it can be hypothesized that propionate may downregulate microglial TNFα production. However, given the demonstrated limitations of the results, further detailed studies are required to shed light on the inflammatory status of microglia. In addition, other cell types need to be considered in future research, such as astrocytes, which have been identified as key players in the inflammatory response and sources of cytokines [73]. Further, the cellular mechanism of propionates effect remains to be elucidated. Next to signaling pathways, including SPI1, other transcription factors are involved in HD-specific neuroinflammatory mediation, such as NFkB signaling and JAK/STAT signaling in microglia and astrocytes, respectively [74].

The impact of IL-6 on HD disease progression remains to be determined, as it appears to have both positive and negative effects. Studies suggest that IL-6 may support the development of HD as an early inflammatory marker [75], while others indicate that a deficiency in IL-6 worsens the clinical course [76]. Tthe exact role of IL-6 in HD remains to be elucidated. Present results do not indicate a dysregulation of IL-6 mRNA in 12-week-old R6/2 mice, neither in the striatum nor in the cortex. Also, propionate treatment did not influence IL-6 mRNA expression. These results suggest a differentially regulated expression of pro-inflammatory cytokine transcription during inflammation in R6/2 mice, potentially involving distinct signaling pathways.

The dosage of 150 mM propionate used in the present study was previously established by Haghikia et al. [42] and corresponds to concentrations already used in humans in MS studies [40]. Following oral intake, propionate is quickly absorbed in the gut and transported via blood flow to the liver and outer peripheral tissues, leading to decreased systemic SCFA concentrations, especially in the brain [36]. The actual concentration of propionate in the CNS after oral supplementation is not known and given the aspect of high bioavailability, only a small fraction will reach the CNS. Still, raising systemic propionate concentration by oral gavage increases propionate’s likelihood of passing the blood-brain barrier and thus exerting effects in the CNS. However, in light of the moderate effects observed, higher dosing strategies might achieve more robust therapeutic outcomes in the context of HD. However, it is important to note that the effects of propionate are dose-dependent and may have adverse effects at excessive concentrations in the CNS, as seen in rats intravenously injected with high dosages propionate worsens depressive-like behavior and dysregulates neurotransmitters such as e.g., dopamine [77].

In summary, this study describes a pro-inflammatory environment in the CNS of 12-weeks-old R6/2 mice, which became evident by elevated iNOS- and TNFα-expression levels. Interestingly, the inflammatory profile in R6/2 mice occurs as a brain region-specific, seen pronounced in the cortex, but less in the striatum. The immune-modulatory effect of propionate in the brains of R6/2 mice became evident by the reduction of IBA1 and iNOS expression, as well as TNFα cytokine and SPI1 mRNA levels. Thus, these findings underline the anti-inflammatory effect of propionate treatment. In line with the inflammatory profile, immunomodulation of propionate was consistent within the cortex. The subsequent impact on disease progression remained moderate and could be demonstrated with only the Clasping score was alleviated in propionate-treated mice. This experimental setup focused on motoric symptom progression and while a reduction in neuroinflammatory markers was observed, the behavior improvements were less pronounced. These findings underline the importance of neuronal protection in HD rather, than solely targeting CNS inflammation. Nonetheless, a potential impact on other HD-related cognitive and psychiatric symptoms warrants further investigation, as neuroinflammation is considered a key driver of these deficits [78,79]. In addition, it has been shown that propionate can ameliorate depressive-like behavior in rats were it enhances grooming behavior, reward-seeking behavior, and activity levels [77].

## 4. Materials and Methods

### 4.1. Mice Genotyping and Treatment

**Figure 6:**
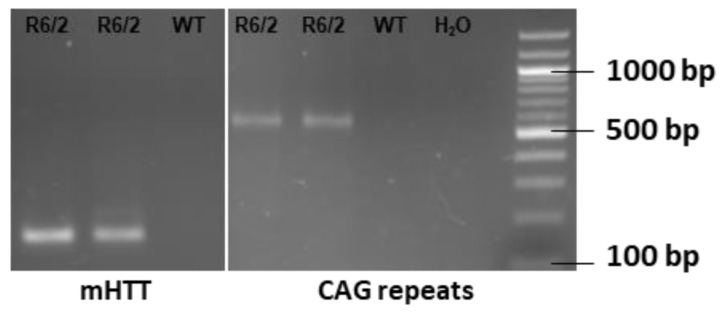
Representative image of genotyping results. Samples of transgenic mice show a band at ~170 bp for mutated Htt and ~600 bp for CAG-repeat, whereas healthy littermates did not show a PCR product. TG = transgene, WT = wild-type, bp = base pairs.

Ovarian-transplanted (OT) hemizygous female mice transgenic for the 5′ end of the human HD gene with a repeat length of approximately 160 ± 5 were purchased from Jackson Laboratories (Bar Harbor, ME, USA, Stock 002810) were bred with male C57BL/6J-mice (Charles River, Köln, Germany) to obtain R6/2 mice of the F1-generation. For further breading heterozygous R6/2-males were backcrossed with C57BL/6J-females. Hemizygous age-matched mice of the F2-generation with a verified CAG-repeat length of 160 ± 5 and both sexes were used. Animals were housed in mixed genotype single-sex cages under standard conditions with a 12-h light/dark cycle at 22 °C with ad libitum access to water and food, fitted with houses and nesting material. Healthy littermates served as the control group. Ear tissue from 15–17 days old mice was obtained to detect expression of mutated Htt as well as CAG-repeat length with polymerase-chain-reaction. DNA was extracted with the KAPA Express Extract (Roche, Basel, Switzerland) and PCR was performed with the KAPA2G fast HotStart genotyping mix (Roche) according to manufacturers’ protocols. Bands were identified at ~170 bp for mutated Htt and ~600 bp for CAG-repeats (Figure 6). Primers, as listed in Table 1, were purchased from Microsynthesis (Balgach, Switzerland).

Age-matched mice were randomly divided into control and treatment groups. The number of male and female mice was thereby randomly distributed in the treatment groups. The treatment group received a daily dose of 150 mM propionate (Sodium propionate, Sigma Aldrich, St. Louis, MI, USA) dissolved in tap water by oral gavage with a blunt end feeding needle—controls received only tap water. Treatment started at the age of 30 days and stopped at 80 days (±2 days), according to the individual pathophysiological appearance. In the survival study, R6/2-mice were gavaged until they died naturally or became moribund. Body weight was measured every second day. All analyses were conducted from two or three independent experiments.

All experiments and protocols were approved by the local authorities for animal experimentation (LANUV, approval ID: 84-02.04.2017.A213), and all methods were carried out in accordance with relevant guidelines and regulations.

### 4.2. Behavioral Analysis

**Figure 7:**
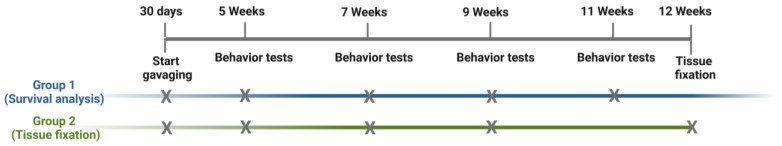
Experimental timeline.

All mice were subjected to behavior testing at the age of 5, 7, and 9 weeks. Mice of survival study accessed tests additionally at 11 and 12 weeks, if physiologically permissible (Figure 7). For all animals, the same testing conditions were granted. Tests were performed in the early afternoon and mice adapted conditions 1 h prior to testing. Motor coordination and balance were tested on a five-station mouse rotarod (Ugo Basile, Biological Research Apparatus, Varese, Italy). Mice were first trained at the age of 4 weeks at a constant speed of 10 rpm with a maximum of 240 s. For experimental testing, settings were changed to an acceleration of 4 to 40 rpm over a maximum period of 240 s. The latency to fall was measured twice (with a minimum pause of 45 min) on the testing day. For dyskinesia progression, hind limb clasping [45] was observed by lifting mice by the tail upside down for a maximum of 45 s about 40 cm above the ground. A three-step scoring system based on time taken to clasp was applied as described earlier [80]. Clasping was rated positive when both hind limbs were touching.

Mice were divided into two experimental groups. In the first group, R6/2 mice underwent survival analysis to assess their lifespan. The second group, both R6/2 mice and wild-type mice were euthanized at approximately 12.5 weeks of age, representing a late stage of HD, to evaluate neurodegeneration and neuroinflammation in brain tissue. Starting at 30 days of age, mice in both groups received daily oral gavage of either 150 mM propionate or tap water. Throughout the study, behavioral tests (rotarod, open field, and clasping score) were conducted at selected time points to monitor motor symptom progression in R6/2 mice. (Created in BioRender).

### 4.3. Tissue Preparation

Mice were i.p. injected with an individual dose (10 μL/g) of narcotic agent containing Ketamine (100 mg/kg, 25%), Xylazine (5 mg/kg, 6.3%) and Vetranquil (5.1 mg/kg, 2.5%) in 0.09% saline. Transcardial perfusion started with saline. For analysis such as Western Blot and rt-PCR brains were snap frozen in liquid nitrogen and afterwards stored at −80 °C. For histological studies, additional perfusion with 4% PFA in 0.1 M PB was performed. Brains were post-fixed in 4% PFA and subsequently stored in 30% sucrose for 24 h prior to embedding in O.T.C. Compound medium (TEK Tissue) and freezing at −20 °C. Brains were cut with a cryostat in 10 µm thick slides.

### 4.4. Immunohistochemistry

For further analysis, the motor cortex and striatum of coronal brain sections in the range of Bregma 1.10 mm to 0.14 mm [81] were selected.

Slides were boiled in citrate buffer (pH 6) for antigen retrieval. After cooling, unspecific binding was prevented by blocking with a mix of 5% bovine serum albumin and 3% normal goat serum in PBS for at least 30 min. Primary antibody (rb anti-Iba1 1:750, Wako/rb anti-NeuN 1:1000, Millipore/rb anti-iNOS 1:50, Enzo Life Science/ms anti-clone mEM48 1:750, Millipore, Burlington, MA, USA) was applied. For fluorescence staining secondary antibodies were incubated for at least 45 min in a 1:1000 dilution. After washing, slides were mounted in DAPI-Fluoromount (SouthernBiotech, Birmingham, AL, USA). For diaminobenzidine (DAB) staining endogenous peroxidase was blocked. Furthermore, sections were incubated with the appropriate biotinylated secondary antibody. Afterward, avidin-biotin-complex (ABC-Kit, Vectastain, Vector Laboratories, Wertheim, Germany) was added for 35 min. DAB (Merck, Rahway, NJ, USA) incubated for 5 min. Tissue was counter-stained with Meyer’s hemalaun solution (VWR, Radnor, PA, USA) and mounted afterwards with mounting medium (Cytoseal XYL, Thermo Scientific, Waltham, MA, USA). Pictures were taken with a light microscope (Olympus BX51, ColorView Soft Imaging System) or fluorescence microscope (Olympus BX51). ImageJ 1.50 software was used for cell counting [82].

### 4.5. Realtime-PCR

Snap-frozen brain tissue was shortly thawed on ice to allow easy cutting. The olfactory bulb and frontal cortex were partly removed until the corpus callosum, and lateral ventricles were visible. Striatum and motor cortex were dissected and stored in trizol. Afterwards, tissue was homogenized with a tissue lyser (TissueLyserII, Qiagen, Hongkong). RNA isolation was performed with Qiagen RNA Lipid Isolation Kit according to the manufacturer’s protocol and concentration was measured with a Nanodrop-1000 spectrophotometer (Peqlab Biotechnology, Erlangen, Germany). RNA was transcribed to cDNA by reverse transcription using SuperScript II Reverse Transcriptase (Invitrogen, Darmstadt, Germany). rtPCR was performed with SYBR Green GoTaq^®^ qPCR Master Mix (Promega, Charbonnières-les-Bains, France) on an Applied Biosystems 7200 Real-time PCR System (Applied Biosystems, Darmstadt, Germany). Duplicates were analyzed and normalized to the geometric average expression of two housekeeping genes (β-actin and 18s ribosome subunit). Primer pairs, as listed in Table 2, were purchased by Microsynthesis (Balgach, Switzerland).

### 4.6. Western Blot

Brain samples were lysed in Trizol using a tissue lyser (TissueLyser II, Qiagen). The protein fraction was separated by adding chloroform, followed by centrifugation at 10,000× *g* for 15 min. The resulting protein pellets were washed twice with 100% ethanol and once with 0.3 M guanidine phosphate buffer. After air drying, the pellets were dissolved in 1% SDS. Protein concentration was determined using the BCA Kit (Thermo Scientific) according to the manufacturer’s protocol. For SDS-PAGE, 20 µg of each sample was loaded. To identify expected molecular weight a pre-stained ladder was used (PageRuler, Thermo Scientific, #26619). Proteins were blotted on a nitrocellulose membrane. Membrane was blocked with 5% milk powder in TBS buffer and afterwards incubated with primary antibody (IBA1, DAKO, 1:300 and GAPDH, Santa Cruz, 1:1000) overnight at 4 °C. Following HRP conjugated secondary antibodies were incubated for 2 h (gt anti-ms IgG, (AP124P) EMD Millipore and gt anti-rb IgG (AP132) Merck). Protein bands were detected by chemiluminescence (Immobion Western HRP, Merck) with an imaging system (ChemiDoc™, BIO RAD, Hercules, CA, USA) and quantified with Image Studio™ light, (LI-COR, Version 5.2).

### 4.7. Statistics

Statistical analysis was performed with GraphPad Prism 8.00 and 9.31 (GraphPad Software, La Jolla, CA, USA). Data from immunohistological and immunofluorescent stainings, Western Blot, and *rt*PCR were statistically tested with one-way analysis of variance (ANOVA) with Bonferroni’s post hoc analysis for multiple testing, if not stated otherwise. Survival analysis was assessed with the Kaplan-Meier analysis with a log-rank test. For behavior tests such as clasping score, rotarod, as well as body weight, differences were examined using two-tailed unpaired Student’s *t*-tests or with two-way ANOVA with Bonferroni’s post hoc analysis for multiple testing, whichever was appropriate. A probability level of * *p* <  0.05, ** *p* <  0.01, ***  *p* <  0.001 was considered to be statistically significant for all tests. The numbers of animals analyzed in each testing group are given as n. Data are shown as mean ± standard error of the mean (SEM).

## Figures and Tables

**Table 1:** Primer sequences for genotyping.

Target Gene	Primer Sequences
mutated Htt	5′-CCG CTC AGG TTC TGC TTT TA-3′ sen
	5′-TGG AAG GAC TTG AGG GAC TC-3′ ase
CAG-repeat	5′-GGC GGC TGA GGA AGC TGA GGA G-3′ sen
	5′-ATG AAG GCC TTC GAG TCC CTC AAG TCC TTC-3′ ase

**Table 2:** Primer sequences for rtPCR.

Target Gene	Primer Sequences
β-Actin	5′ CCT CTA TGC CAA CAC ACT GC sen
	5′ CAT CGT ACT CCT GCT TGC TG ase
18S ribosomal RNA	5′ GTA ACC CGT TGA ACC CCA TT sen
	5′ CCA TCC AAT CGG TAG TAG ase
IL-6	5′ CCG GAG AGG AGA CTT CAC AG sen
	5′ GGA AAT TGG GGT AGG AAG GA ase
TNFα	5′ GCC TCT TCT CAT TCC CGC TT sen
	5′ CTG ATG AGA GGG AGG CCA TT ase
BDNF	5′ CTT ATG AAT CGC CAG CCA ATT CTC sen
	5′ TGC AGG GGC ATA GAC AAA AGG ase

## Data Availability

The raw data supporting the conclusions of this article will be made available by the authors on request.
